# Design and Optimization of a Novel Hybrid Membrane–Electrochemical Hydrogen Pump Process for Recovering Helium from NRU off Gas

**DOI:** 10.3390/membranes13070689

**Published:** 2023-07-24

**Authors:** Wu Xiao, Hao Wang, Andi Cheng, Hanli Wang, Zhendong Yang, Xuemei Wu, Xiaobin Jiang, Gaohong He

**Affiliations:** 1 State Key Laboratory of Fine Chemicals, Frontier Science Center for Smart Materials, Dalian University of Technology, Dalian 116024, China; wuxiao@dlut.edu.cn (W.X.); haow@mail.dlut.edu.cn (H.W.); adcheng@mail.dlut.edu.cn (A.C.); xuemeiw@dlut.edu.cn (X.W.); xbjiang@dlut.edu.cn (X.J.); 2 Shandong Huaxia Shenzhou New Material Co., Ltd., Zibo 256401, China; whl89333@163.com (H.W.); yangzhendong@huaxiashenzhou.com (Z.Y.)

**Keywords:** hybrid process, helium separation, process design, economic assessment, optimization, response surface methodology

## Abstract

Due to the low boiling point of helium, the nitrogen-rich off gas of the nitrogen rejection unit (NRU) in the liquefied natural gas (LNG) plant usually contains a small amount of CH_4_, approximately 1–4% He, and associated gases, such as H_2_. However, it is difficult to separate hydrogen and helium. Here, we propose two different integrated processes coupled with membrane separation, pressure swing adsorption (PSA), and the electrochemical hydrogen pump (EHP) based on different sequences of hydrogen gas removal. Both processes use membrane separation and PSA in order to recover and purify helium, and the EHP is used to remove hydrogen. The processes were strictly simulated using UniSim Design, and an economic assessment was conducted. The results of the economic assessment show that flowsheet #2 was more cost-effective due to the significant reduction in the capacity of the compressor and PSA because of the pre-removal of hydrogen. Additionally, using the response surface methodology (RSM), a Box–Behnken design experiment was conducted, and an accurate and reliable quadratic response surface regression model was fitted through variance analysis. The optimized operating parameters for the integrated process were determined as follows: the membrane area of M101 was 966.6 m^2^, the permeate pressure of M101 was 100 kPa, and the membrane area of M102 was 41.2 m^2^. The maximum recovery fraction was 90.66%, and the minimum cost of helium production was 2.21 $/kg. Thus, proposed flowsheet #2 has prospects and value for industrial application.

## 1. Introduction

Helium is a unique and valuable chemically inert gas because of its low boiling point (−269 °C), low solubility, and excellent diffusivity [1]. It is widely utilized in various fields such as the military, aerospace, semiconductor, deep-sea diving, and medical applications. Due to the extremely low concentration of helium gas in the air, which is only 0.0005 mol% [2], the extraction of helium from natural gas is currently almost the sole industrial source of helium [3].

Helium is typically produced as a byproduct of liquefied natural gas (LNG) production. The off gas from the nitrogen rejection unit (NRU) in LNG plants contains approximately 1–4% helium [3,4]. The helium in the off gas is recovered and purified through a cryogenic distillation process, but this conventional helium recovery method requires high investment and energy consumption [5,6]. Due to the similar molecular size and similar boiling points of hydrogen and helium, membrane processes and adsorption processes widely used in hydrogen separation [7,8] can also be applied to helium recovery and purification processes [9]. These processes offer alternative options for helium recovery that are more cost-effective and energy-efficient [10,11,12,13]. Hamedi et al. [14] proposed a helium recovery process based on highly selective silicon dioxide membranes. Their research indicates that, compared to conventional multistage polymer membranes, single-stage silicon dioxide membranes are more economical because of the elimination of interstage compression. Scholes et al. [15] conducted a techno-economic study on the membrane separation process for helium recovery from natural gas and NRU off gas. The results indicate that membrane separation becomes economically competitive when the helium concentration in natural gas is ≥0.3 mol%. For the recovery and purification of helium from the NRU off gas, a combined membrane-pressure swing adsorption (PSA) process is more cost-effective compared to the conventional cryogenic distillation process.

However, the similar molecular sizes and similar boiling points of hydrogen and helium make it challenging to achieve their separation using conventional gas separation methods, such as membrane, cryogenic distillation, and PSA. Currently, the industrial removal of hydrogen is primarily achieved through catalytic oxidation—converting hydrogen into water, which is then separated [16]. However, this process inevitably introduces new impurities such as O_2_, leading to increased energy consumption and a waste of hydrogen resources. Electrochemical hydrogen pump (EHP) technology, driven by electrical energy, offers an effective solution to overcome the challenge posed by the similar properties of hydrogen and helium [17,18]. Hydrogen is selectively transported as protons through the membrane electrode assembly, allowing for the one-step separation of hydrogen and helium. Onda et al. [19] used the EHP to recover hydrogen from H_2_/CO_2_ or H_2_/N_2_ mixed gases. After processing, the released anode gas had a hydrogen concentration of <50 ppm. Nordio et al. [20] successfully used the EHP to recover hydrogen from the H_2_/He mixture. When the feed gas had a helium content of 20%, the H_2_ purity of cathode was 99.97%. The EHP caused almost no loss of helium. However, the application of the EHP for hydrogen–helium separation is currently limited to the laboratory stage. For real gases with complex components, such as NRU off gas, it is hoped that novel technologies, such as the EHP, could be widely implemented in engineering practice to achieve efficient hydrogen–helium separation.

In this work, our objective is to design a novel process for helium recovery from the NRU off gas by combining membranes, PSA, and EHP. We designed two different coupling separation processes based on different sequences of hydrogen gas removal. A sensitivity analysis was conducted for the optimization of both two processes to achieve the production target of an 80% recovery fraction. The preferred process was determined through an economic assessment. Additionally, the key operational parameters of the preferred process, such as the membrane area and membrane permeate pressure, were optimized using response surface methodology (RSM).

## 2. Method

### 2.1. Feed Conditions

The overhead nitrogen-rich gas stream from the NRU in an LNG plant typically contains a small amount of CH_4_, approximately 1–4% He, and associated components, such as H_2_, Ne, etc. [3,4]. Based on the NRU simulation results from Quader’s study [4], we determined a feed flow rate of 76 kmol/h. Since the original simulation did not consider the hydrogen component, we adapted the NRU tail gas composition by adding hydrogen to it. The feed conditions and composition for this study are shown in Table 1.

### 2.2. Process Configurations

**Figure 1:**
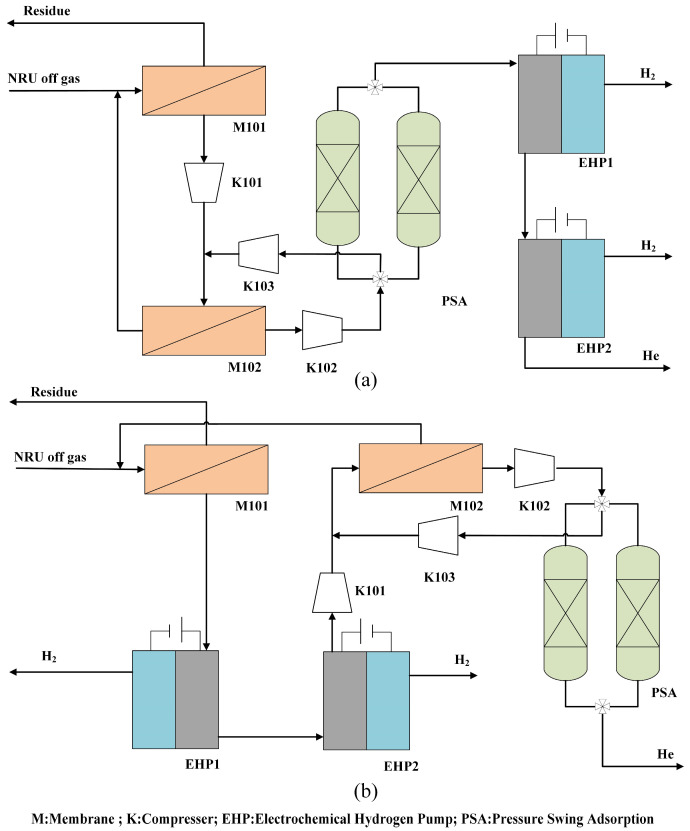
The coupling process flowsheets: (**a**) flowsheet #1; (**b**) flowsheet #2.

Process flowsheet #1 is shown in Figure 1a. The off gas from the NRU is mixed with the residue recycle from the second-stage membrane M102, and the mixed stream feeds the first-stage membrane M101.The permeate from M101 is recompressed and fed into the second-stage membrane M102 for further enrichment. The permeate gas from M102 is helium-rich stream. The helium-rich stream then passes through the PSA unit to remove impurities, such as N_2_ and CH_4_. The gas stream, after the PSA process, enters EHP1. Hydrogen is fully removed by the two-stage EHP. Anode residue gas from EHP2 is the helium product. Due to the high concentration of helium in the desorption gas from the PSA unit, the desorption gas is recycled to the feed of the second-stage membrane for further helium recovery.

As shown in Figure 1b, process flowsheet #2 is similar to #1. However, the permeate gas from M101 now directly enters the two-stage EHP for the complete removal of hydrogen. The anode residue gas from EHP2 is recompressed and fed into M102. The permeate gas from M102 enters the PSA unit to remove impurities, such as N_2_ and CH_4_. The gas stream, after the PSA process, is the helium product. The residue gas from the second-stage membrane M102 and the desorption gas from the PSA unit are recycled in the same manner as in process flowsheet #1.

### 2.3. Simulation Methodology

The processes were simulated using the Peng–Robinson fluid properties package in UniSim Design (Honeywell, Charlotte, NC, USA). Currently, UniSim Design does not integrate a simulation model for membrane separators. Our research group developed a membrane module based on the numerical algorithm proposed by Chen et al. [21]. to simulate gas membrane separation processes. In the simulation, the membrane module used was a hollow fiber membrane contactor. The feed gas and permeate gas flowed in a counter-current pattern. Our previous works [21,22] verified the accuracy of this model.

Indeed, the single-stage silica membrane process, as introduced in the introduction, is more economical due to the elimination of interstage compression. However, the fragility of the silica membrane remains an issue. Additionally, the complex preparation process and control conditions of the silica membrane limit the feasibility of large-scale commercialization. In contrast, commercial polyimide, as a polymer membrane material, has been widely used in the field of hydrogen separation and its cost is relatively low, making it more suitable for helium separation applications.

Here, we selected commercial polyimide as the gas separation membrane material. The performance parameters of the membrane material are shown in Table 2 [23]. These gas separating properties were supplied by Permea China Ltd., Yantai, China, the subsidiary of Air Products and Chemical, Inc., Allentown, PA, USA.

The simulation of the electrochemical hydrogen pump module was conducted using a partial element stage cut EHP model that was independently developed by our research group [24]. This model takes into account three real factors: anode impurity diffusion, hydrogen back-diffusion, and anode catalyst deactivation. By establishing real-time communication between the Python EHP model and UniSim Design, the rapid prediction of the hydrogen separation performance of the EHP can be achieved.

Similarly, there is a lack of a strict simulation model for PSA in UniSim Design, but simple simulations can be conducted using the component splitter provided by UniSim Design. The PSA system adopts the PSA design proposed by Weh et al. [25]. The adsorbent used is zeolite 13X, which adsorbs N_2_ and CH_4_. The feed pressure for the PSA is 500 kPa, and the desorption pressure is 140 kPa. The split fractions for various components in the PSA system were calculated as shown in Table 3.

## 3. Sensitivity Analysis and Flowsheet Economic Comparison

### 3.1. Impacts of the Membrane Area on the Purity and Recovery of Helium

**Figure 2:**
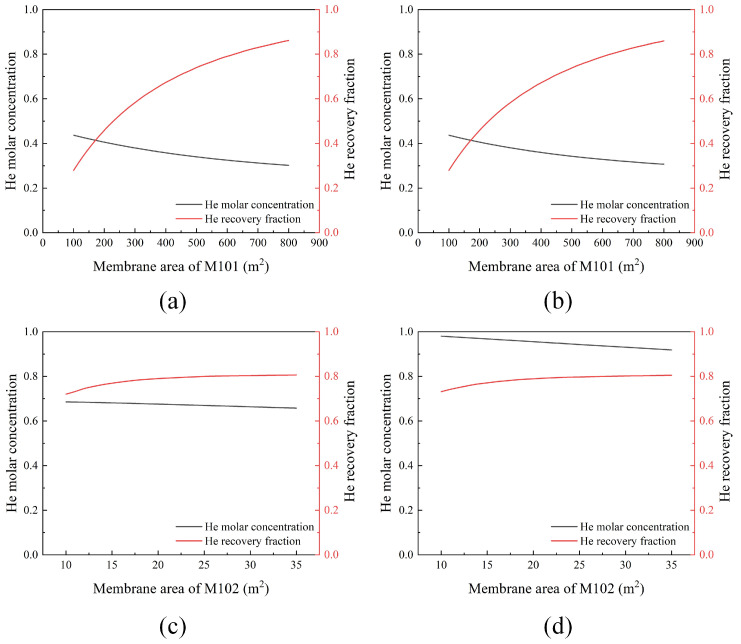
Effects of the membrane area on the He purity and recovery fraction under constant permeate pressure. (**a**) Membrane area of M101 in flowsheet #1; (**b**) membrane area of M101 in flowsheet #2; (**c**) membrane area of M102 in flowsheet #1; (**d**) membrane area of M102 in flowsheet #2.

The membrane is the core component of the membrane module, and the membrane area dictates the investment and separation performance of the membrane module. The influences of the membrane area on the helium purity and recovery fraction are shown in Figure 2. It can be observed that the recovery of helium increases but the purity of helium decreases with the increase in the membrane area. There is a certain trade-off effect between the recovery fraction and purity. Furthermore, as the membrane area increases, the increasing trend in the recovery fraction gradually levels off. Based on Figure 2 and separation necessity, the two membrane areas optimized for flowsheet #1 are 630 and 30 m^2^, respectively. The area of flowsheet #2 remains the same as in flowsheet #1. 

### 3.2. Impacts of the Permeate Pressure on the Purity and Recovery of Helium

**Figure 3:**
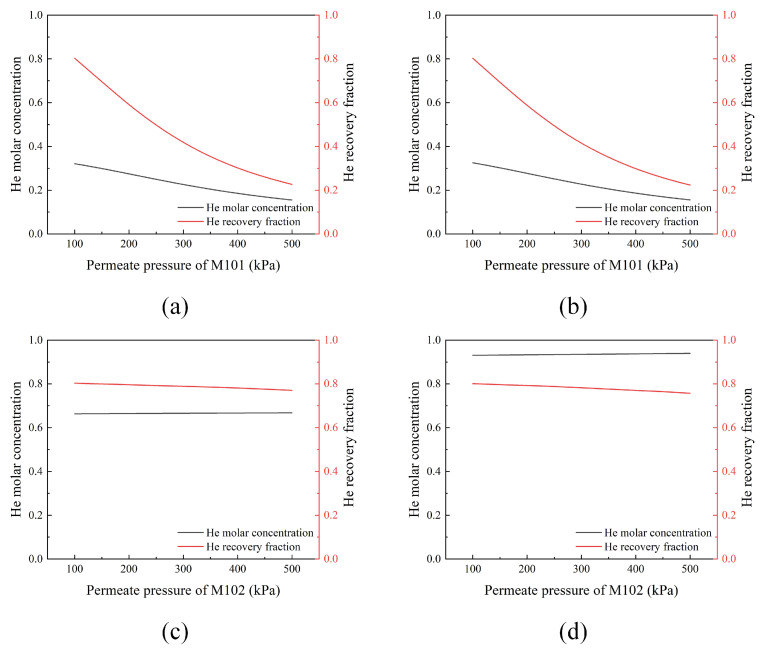
Effects of the permeate pressure on the He purity and recovery fraction with a constant membrane area. (**a**) Permeate pressure of M101 in flowsheet #1; (**b**) permeate pressure of M101 in flowsheet #2; (**c**) permeate pressure of M102 in flowsheet #1; (**d**) permeate pressure of M102 in flowsheet #2.

The pressure ratio on both sides of the membrane, known as the feed permeate pressure ratio, is one of the main driving forces in gas separation membrane processes [26]. It affects the flow rate and composition of permeate gas. Increasing the feed permeate pressure ratio can effectively enhance the separation performance of the membrane. However, it can also increase the interstage compression costs. In this work, in order to reduce the number of compressors, we set the feed pressure to 2500 kPa to be constant and varied the permeate pressure to change the pressure ratio. The impacts of the permeate pressure on the helium purity and recovery fraction are shown in Figure 3. As the permeate pressure increases, the pressure ratio decreases, and the recovery fraction also decreases. According to Figure 3 and product specification, 100 kPa was selected as the permeate pressure for the membrane processes, resulting in a feed permeate pressure ratio of 25, which meets the recovery target of 80%.

### 3.3. Impacts of the Applied Potential on the Recovery Fraction of H_2_ and the Energy Efficiency

The applied potential of EHP is a key factor that affects the performance of the EHP [27]. The applied potential provides energy for the electrochemical reaction of hydrogen, directly influencing the energy efficiency and overall energy consumption of the EHP. The current density of the hydrogen pump follows Faraday’s law, which allows us to calculate the hydrogen recovery fraction based on this relationship, as defined in Equation (1). Additionally, energy efficiency is another important parameter that directly reflects the energy consumption of the EHP. It is defined as shown in Equation (2).

(1) $$R=\frac{j_{ave}A}{2FF_{H_{2}}^{in}}$$

(2) $$\eta =\frac{\frac{j_{ave}A}{2F}\Delta H_{c}-j_{ave}AV}{F_{H_{2}}^{in}\Delta H_{c}}$$

where $j_{ave}$ is the average current density (A·m^−2^); A is the Membrane Electrode Assembly (MEA) area (m^2^); F is the Faraday constant (96,485 C·mol^−1^); $F_{H_{2}}^{in}$ is the flow rate of H_2_ in the feed (mol·s^−1^); and $\Delta H_{c}$ is the combustion heat of H_2_ (285.8 kJ/mol).

**Figure 4:**
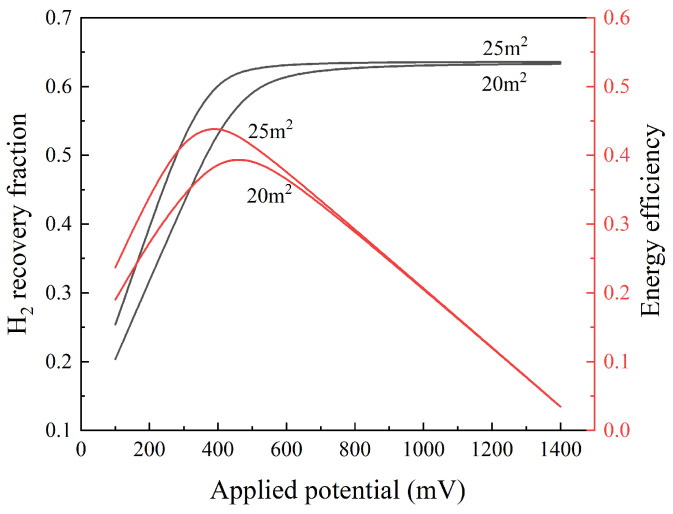
Effects of the applied potential of EHP1 on the H_2_ recovery fraction and energy effciency.

When the MEA area is constant, the applied potential also has an impact on the hydrogen recovery fraction. Taking EHP1 as an example, it can be observed from Figure 4 that when the MEA area is sufficient, the applied potential becomes a controlling factor that affects the performance of the EHP. As the applied potential increases, the hydrogen recovery fraction also increases. The energy efficiency of the EHP initially increases with the applied potential but reaches a peak value and then decreases as the applied potential continues to increase. To reduce the energy consumption of the EHP, 500 mV was selected as the applied potential for the EHP unit.

### 3.4. Impacts of the MEA Area on the Recovery Fraction of Hydrogen

**Figure 5:**
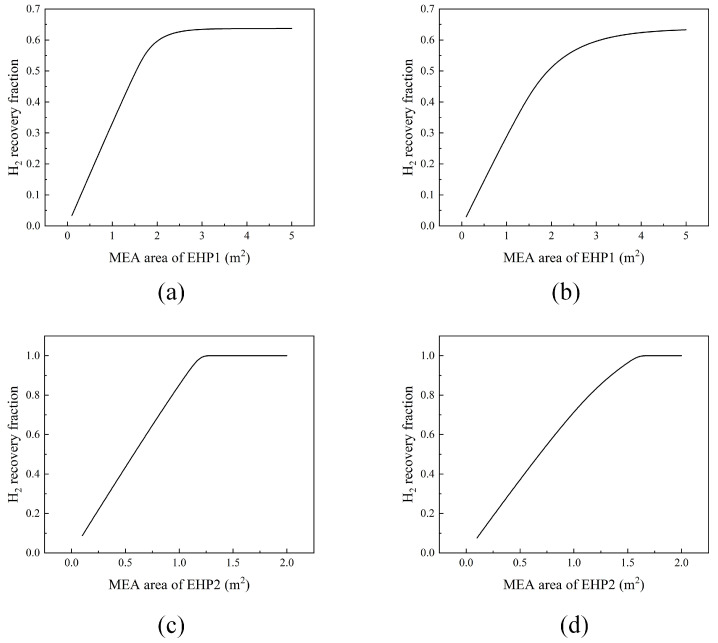
Effects of the MEA area on the H_2_ recovery fraction. (**a**) MEA area of EHP1 in flowsheet #1; (**b**) MEA area of EHP1 in flowsheet #2; (**c**) MEA area of EHP2 in flowsheet #1; (**d**) MEA area of EHP2 in flowsheet #2.

The MEA is the core component of the EHP, and the MEA area reflects the scale of the EHP. It includes major investment components such as a platinum–carbon catalyst, gas diffusion layer, and proton exchange membrane. Figure 5 shows the impact of the MEA area on the hydrogen recovery fraction. Under a certain applied potential, the hydrogen recovery fraction increases with the increase in the MEA area and eventually levels off. To achieve the complete removal of hydrogen, the MEA areas of EHP1 and EHP2 in flowsheet #1 are 2.5 and 1.2 m^2^, respectively. The MEA areas in flowsheet #2 are 4 and 1.6 m^2^, respectively.

### 3.5. Economic Comparison

The main difference between the two processes lies in the sequence of hydrogen removal. In flowsheet #1, hydrogen is removed last to reduce the processing capacity required for the EHP. In contrast, flowsheet #2 chooses to remove hydrogen earlier to decrease the processing capacity required for the compressors and the PSA unit. In this work, the cost of helium production was chosen as the evaluation index to analyze the economics of the above two simulated processes and compare the economics of the two processes. The cost parameters of the economic evaluation are shown in Appendix A.

According to Table 4, in the helium recovery and purification process from the NRU off gas, membrane separator investment and PSA investment are the main contributors to the total investment (60–70%). Due to the pre-removal of hydrogen in flowsheet #2, the investments in compressors and PSA units are significantly reduced, while the increase in hydrogen pump investment is minimal. As a result, the total investment cost is greatly reduced. The total investment in flowsheet #2 is 445.56 thousand $, which is 42.93 thousand $ less than that in flowsheet #1. The helium production costs of flowsheets #1 and #2 are 2.05 and 1.92 $/kg He, respectively. Therefore, flowsheet #2 is a more cost-effective process design.

## 4. Optimization of Flowsheet #2 Based on Response Surface Methodology

In complex process simulations, there is often a trade-off relationship between the quality of the target product and the production cost. For example, increasing the membrane area improves the separation performance but also increases the investment cost of the membrane module. In addition, there are strong interactions between operating parameters in the process. For instance, the membrane area of M101 determines the feed flow rate and composition of M102, thereby affecting the M102 area under the same separation requirement. At the same time, because the residue of M102 returns to the inlet of M101 to recycle helium, the area of M101 is affected by the area of M102. The interactions cause the variables in the coupled system to affect each other.

Response surface methodology (RSM) has been used to explore, optimize, and model the performance of complex systems. RSM is a combination of mathematical and statistical methods. By modeling the relationship between variables and responses, it can effectively optimize multiple variables at the same time [28,29,30]. This method is actually a polynomial approximation of the effective variables and their interactions in an unknown complex model. Based on the results of the sensitivity analysis, the optimal range of influential parameters was determined, and then the statistical method RSM was used for experimental design and process optimization.

For preferred flowsheet #2, multiobjective optimization was performed to achieve the maximum helium recovery fraction and the minimum production cost. Based on sensitivity analysis of Section 3.1 and Section 3.2, three significant variables were selected, the membrane area of M101, the permeate pressure of M101, and the membrane area of M102, which serve as response functions for the helium recovery fraction and cost of helium production. The ranges of optimization variables are shown in Table 5. A quadratic regression model was fitted using the Box–Behnken Design (BBD) and then optimized. Table 6 represents the 17 experiments designed based on the BBD method using Design Expert, including four repeated experiments. According to the simulation results from Table 6, the quadratic polynomial regression equation models, expressed in terms of coded factors, are as follows:

$$RF=69.38+14.32A-14.69B+4.68C+1.53AB+1.49AC+0.93BC-4.04A^{2}\;+0.3537B^{2}-3.61C^{2}$$

$$PC=2.35+0.2225A+0.4275B-0.1925C-0.1075AB-0.1075AC-0.0725BC\;+0.0687A^{2}+0.0887B^{2}+0.1887C^{2}$$

where RF is the recovery fraction of helium; PC is the cost of helium production ($/kg He) A is the membrane area of M101 (m^2^); B is the permeate pressure of M101 (kPa); and C is the membrane area of M102 (m^2^).

The analysis of variance (ANOVA) of the data was applied to analyze the fitness and adequacy of the experimental model, as shown in Table 7.

As can be seen from Table 7, whether it is for RF or PC, the F-values of 654.80 and 115.24 imply that the models are significant. There is only a 0.01% chance that a “Model F-value” could occur because of noise. Both *p*-values are less than 0.0001, indicating that the models are statistically significant at the 95% confidence level. The regression model’s coefficients $R^{2}$ of the regression model are 0.9988 and 0.9933, respectively. The closer $R^{2}$ is to 1, the better the data fit the model. The predicted correlation coefficients $R_{pred}^{2}$ are basically consistent with the adjusted correlation coefficients $R_{adj}^{2}$ The differences are less than 0.2. Additionally, the variation coefficients are only 1.20% and 1.95%, indicating a sufficiently high level of reliability. In summary, the response surface correlation model is in good agreement with the actual situation, can effectively predict and analyze the process, and is suitable for the optimization of key variables.

**Figure 6:**
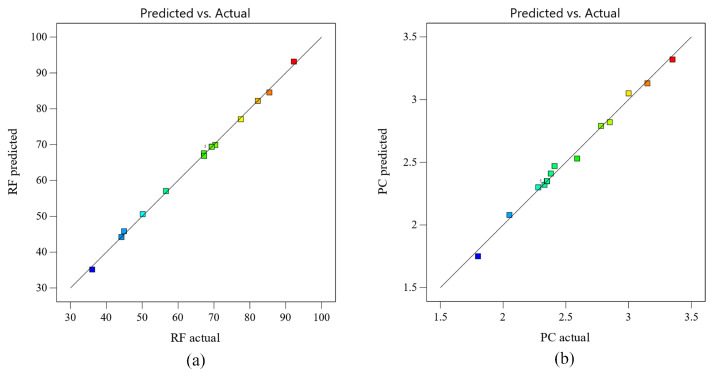
Comparison between the predicted value and actual value. (**a**) RF; (**b**) PC.

The model validation results are shown in Figure 6. From the comparison between the model’s predicted value and the actual value, it can be seen that the model’s predicted value is reasonably consistent with the actual value, and the predicted value falls on a straight line as much as possible. The reliability and accuracy of the regression model are verified again, and it can be used for the prediction and analysis of key parameter optimization.

**Figure 7:**
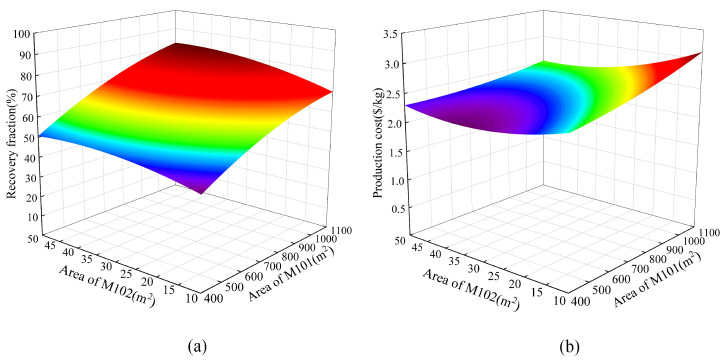
The responses to the area of M101 and M102. (**a**) RF; (**b**) PC.

**Figure 8:**
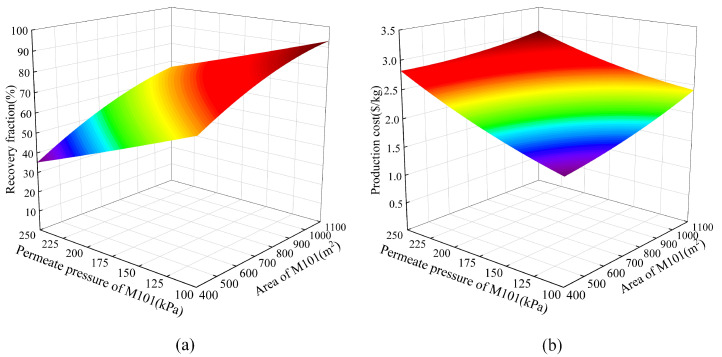
The responses to the permeate pressure and area of M101 (**a**) RF; (**b**) PC.

**Figure 9:**
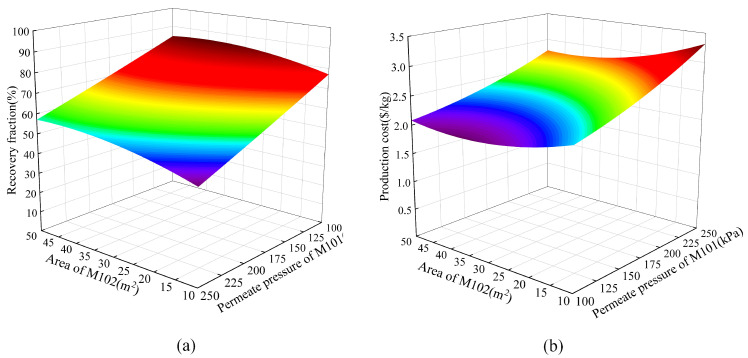
The responses to the permeate pressure of M101 and area of M102. (**a**) RF; (**b**) PC.

According to the fitted response surface model, the corresponding three-dimensional response plots were generated, as shown in Figure 7, Figure 8 and Figure 9. Figure 7 shows the responses of the helium recovery fraction and production cost to the area of M101 and area of M102. It can be observed that as the area of M101 and the area of M102 increase, both the helium recovery fraction and production cost also increase. However, their increasing trends are not exactly the same. When the M102 area is relatively large, such as 30 m^2^, the recovery fraction increases rapidly with the increase in the M101 area, but the production cost does not increase significantly. On the other hand, when the M102 area is small, such as 15 m^2^, the recovery fraction does not increase significantly with the increase in the M101 area, but the production cost increases sharply. Based on this response surface graph, it can be concluded that in order to achieve a higher recovery fraction and lower production cost, the area of M102 should be larger than 30 m^2^.

Figure 8 shows the responses of the helium recovery fraction and production cost to the permeate pressure and area of M101. It can be observed that as the area of M101 increases, both the helium recovery fraction and production cost increase. However, with the increase in the permeation pressure, the helium recovery fraction continuously decreases, while the production cost sharply increases. This is mainly due to the increase in the permeation pressure, which leads to a decrease in the pressure ratio on both sides of the membrane. The decrease in the driving force of the gas membrane separation process leads to a reduction in helium gas production, resulting in an increase in the production cost. Based on this response surface graph, it can be concluded that in order to achieve a higher recovery fraction and lower production cost, the permeate pressure of M101 should be as low as possible, such as 100 kPa.

Figure 9 shows the responses of the helium recovery fraction and production cost to the permeate pressure of M101 and area of M102. It can be observed that the responses to the permeate pressure of M101 in Figure 9 are similar to those in Figure 8, and the responses to the area of M102 in Figure 9 are similar to those in Figure 7.

According to the quadratic response surface regression models, the optimal solution was obtained using Design Expert software, as shown in Table 8. The optimized variables were simulated and calculated again in UniSim Design. the MEA areas of EHP1 and EHP2 were adjusted to 5 and 2 m^2^ to remove all hydrogen. It was found that the maximum He recovery fraction and minimum production cost were 90.66% and 2.21 $/kg He, respectively. Compared to the production cost of 1.92 $/kg mentioned in Section 3.5, despite the increase in cost of 0.29 $/kg, the helium recovery fraction increased by 10.66%. Helium was successfully recovered to a greater extent.

## 5. Discussion

### 5.1. Economic Comparison with the Open Literature

An economic comparison of the helium recovery processes, as mentioned in some open literature, is shown in Table 9. Due to variations in the feed composition, scale, separation objectives, economic parameters, etc., the cost range of helium recovery processes is wide, ranging from 0.65 to 3.3 $/kg helium. However, the overall, hybrid membrane process has certain economic competitiveness for industrial applications and can be used as an alternative to cryogenic distillation.

### 5.2. The Impact of the Membrane Material Selectivity on the Economy

Another commercial Hyflon AD60X membrane with a lower He/N_2_ selectivity was selected as the gas separation membrane to discuss the impact of membrane material selectivity on the economy. The performance parameters of the hollow fiber Hyflon AD60X membrane are shown in Table 10 [33].

As known, there is generally a trade-off between permeability and selectivity. The He/N_2_ selectivity of the Hyflon AD60X membrane is 50.3, and its permeation rate is 309 GPU. The simulation results indicate that, despite the decrease in membrane selectivity, the increase in permeability reduced the total membrane area to 500 m^2^, resulting in a cost reduction of 0.4 $/kg for helium production.

A techno-economic study was conducted by Scholes at al. [15] for the membrane process that recovered helium from the NRU off gas. The results indicate that when the membrane’s He/N2 selectivity is greater than 25, the production cost of helium is competitive against the current market price. This work once again demonstrates this point.

### 5.3. The Size of the Helium Recovery Unit

As a simulation of a large-scale industrial installation, the size of the equipment is also important. Due to the extremely low concentration of helium in natural gas, the helium recovery unit is typically located downstream in LNG plants. Therefore, the footprint of the helium recovery unit in LNG plants is typically very small. 

For instance, a hollow fiber module with a length of 1 m, filled with fibers with a diameter of 100 μm, will have a membrane area of approximately 300 m^2^ [35]. Therefore, the size of helium recovery unit is not typically very large. In this work, the use of approximately three hollow fiber membrane modules with lengths of 1 m is sufficient to achieve the separation objective. 

## 6. Conclusions

In this study, two different integrated processes for recovering and purifying helium from NRU off gas were proposed based on different sequences of hydrogen gas removal. The processes were strictly simulated using UniSim Design, and an economic assessment was conducted. The results of the economic assessment show that flowsheet #2 was more cost-effective due to the significant reduction in the processing capacity of the compressor and PSA because of the pre-removal of hydrogen. Additionally, the optimization of flowsheet #2 was achieved by using response surface methodology. A Box–Behnken design experiment was conducted, and an accurate and reliable quadratic response surface regression model was fitted through the analysis of variance. The optimized operating parameters for the integrated process were determined as follows: the membrane area of M101 is 966.6 m^2^, the permeate pressure of M101 is 100 kPa, and the membrane area of M102 is 41.2 m^2^. The maximum recovery fraction achieved was 90.66%, and the minimum cost of helium production was 2.21 $/kg. Based on an economic comparison with the open literature, the hybrid membrane process has a certain level of economic competitiveness for industrial application and can be used as an alternative to cryogenic distillation.

## Figures and Tables

**Table 1:** Feed data and product requirements.

Stream	Item	Value
Feed	Temperature [K]	313.15
	Pressure [kPa, absolute]	2500
	Flow rate [kmol/h]	76
	Composition [mol%]	
	CH_4_	4.34
	N_2_	90.69
	He	3.47
	H_2_	1.50
Product	He purity	99.999 mol%
	He recovery	80%

**Table 2:** Separation performance parameters of the membrane separator.

Component	Permeation Rate [GPU ^1^]
CH_4_	2.1
N_2_	2.2
He	200
H_2_	210

^1^ GPU = 10^−6^ cm^3^(STP)·cm^−2^·s^−1^·cmHg^−1^.

**Table 3:** Separation performance parameters of the PSA unit.

Component	Split Fractions
CH_4_	1 × 10^−4^
N_2_	1 × 10^−4^
He	0.95
H_2_	0.95

**Table 4:** Summary of the economic assessment data.

Item	Value	
	Flowsheet #1	Flowsheet #2
Capital cost (thousand $)		
Membranes	188.57	188.57
Compressors	40.61	37.89
PSA	142.88	110.68
EHP	3.70	5.60
Other equipment	112.73	102.82
Total investment	488.49	445.56
Depreciation cost (thousand $/a)	78.00	71.46
Operation cost (thousand $/a)		
Electricity	57.32	55.28
Cooling water	4.66	4.54
Total operation cost	61.98	59.82
Total annual cost (thousand $/a)	139.98	131.28
Production cost ($/kg He)	2.05	1.92

**Table 5:** Optimization variables and their ranges.

Variables	Minimum	Center	Maximum
membrane area of M101(m^2^)	400	750	1100
permeate pressure of M101(kPa)	100	175	250
membrane area of M102(m^2^)	10	30	50

**Table 6:** Box–Behnken design and simulation values.

Run	Area of M101 (m^2^)	Permeate Pressure of M101 (kPa)	Area of M102 (m^2^)	Recovery Fraction (%)	Cost of Helium Production ($/kg He)
1	750	175	30	69.38	2.35
2	750	100	50	85.45	2.05
3	400	100	30	67.17	1.80
4	750	175	30	69.38	2.35
5	750	175	30	69.38	2.35
6	750	100	10	77.5	2.33
7	1100	250	30	67.27	3.00
8	400	175	10	44.21	2.41
9	750	250	50	56.61	2.78
10	400	175	50	50.13	2.28
11	1100	100	30	92.27	2.38
12	750	250	10	44.94	3.35
13	1100	175	10	70.36	3.15
14	1100	175	50	82.22	2.59
15	750	175	30	69.38	2.35
16	750	175	30	69.38	2.35
17	400	250	30	36.06	2.85

**Table 7:** The analysis of variance of the correlation model.

Name	Value		Name	Value	
	*RF*	*PC*		*RF*	*PC*
F value	654.80	115.24	$R^{2}$	0.9988	0.9933
*p*-value	<0.0001	<0.0001	$R_{adj}^{2}$	0.9973	0.9847
Mean	65.95	2.51	$R_{pred}^{2}$	0.9810	0.8927
C.V.%	1.20	1.95	Adeq Precision	95.5517	41.7848

**Table 8:** Optimization variables’ values in the system.

Item	Value
membrane area of M101 (m^2^)	966.6
permeate pressure of M101 (kPa)	100
membrane area of M102 (m^2^)	41.2

**Table 9:** Economic comparison of the cost of helium production.

Process Description	He in Feed (mol%)	He Purity (mol%)	He Recovery (%)	Cost ($/kg He)	Ref.
BOC helium refining and liquefaction facility (Darwin, Australia) for NRU off gas	3	99.999	-	3.3	[31]
Four-stage membrane process for He recovery from NRU off gas ^1^	1	90	99	1.77	[32]
Three-stage membrane process integrated with the nitrogen rejection unit for He recovery from nature gas ^2^	3.54	99	91.31	0.65 ^3^	[4]
This work	3.47	99.999	90.66	2.21	

^1^ The feed does not contain any hydrogen components, and the removal of hydrogen is not considered. ^2^ The feed does not contain any hydrogen components, and the removal of hydrogen is not considered. This process uses the noncommercial PBO-co-PPL membrane with a helium permeability of 1105 Barrer and an He/N_2_ selectivity of 61.34. ^3^ Only the helium recovery unit was taken into consideration. The investment of compressors was calculated using the methodology described and the data shown in Table A1 in this work.

**Table 10:** Separation performance parameters of the hollow fiber Hyflon AD60X membrane.

Component	Permeation Rate ^1^ [GPU]
CH_4_	1.95
N_2_	6.15
He	309
H_2_	122

^1^ The performance parameters of the hollow fiber Hyflon AD60X membrane were calculated using the correlation between the permeation rate and permeability coefficient, as described in the literature [34].

## Data Availability

The data presented in this study are available on request from the corresponding author.

## Appendix A. Cost Calculation Method

This part mainly introduces the cost calculation method of the coupling process for helium recovery and purification. The unit operation time was calculated to be 8000 h/a.

The annual equipment depreciation cost includes compressors, membrane separators, a PSA unit, EHPs, and other equipment, which were calculated as shown in Table A1.

**Table A1:** Calculation basis of the annual equipment depreciation cost.

Item	Unit	Price	Depreciation Year
Compressor	$/kW	857.14	15
membrane	$/m^2^	285.7	5
EHP	$/m^2^	1000	2.5
PSA	$	Equation (A1)	5
Other equipment ^1^	$	30% of the main equipment ^2^ cost	15

^1^ Other equipment includes mixers, pipes and valves, control systems, and flash tanks. ^2^ The main equipment includes compressors, membrane separators, EHPs, and PSA.

The PSA investment cost can be calculated by Equation (A1) [36]:

(A1) $$I_{2}=I_{1}\times {\left(\frac{Q_{2}}{Q_{1}}\right)}^{n}$$

where n = 0.75, $I_{1}$, and $Q_{1}$ represent the inherent investment and processing capacity of the PSA device, respectively. $I_{2}$ and $Q_{2}$ stand for the inherent investment and processing capacity of the PSA device after transformation. The original data came from the literature [37].

The annual operating cost includes utilities such as electricity, circulating water, freshwater, saturated steam, and natural gas, and its calculation basis is shown in Table A2.

**Table A2:** Calculation basis of the annual operating cost.

Utilities	Unit Price
Electricity	0.1 $/kW·h
Cooling water	0.071 $/t

Then, the total investment (TI) of helium recovery and purification from NRU off gas can be calculated by Equation (A2):

(A2) $$TI=I_{com}+I_{mem}+I_{PSA}+I_{EHP}+I_{other}$$

where TI is the total investment in thousand $; $I_{com}$ is the compressor investment in thousand $; $I_{mem}$ is the membrane separator investment in thousand $; $I_{PSA}$ is the PSA investment in thousand $; $I_{EHP}$ is the EHP investment in thousand $; and $I_{other}$ is the other equipment investment in thousand $.

The depreciation cost (*DC*) of helium recovery and purification can be calculated by Equation (A3):

(A3) $$DC=D_{com}+D_{mem}+D_{PSA}+D_{EHP}+D_{other}$$

where DC is the depreciation cost in thousand $/a; $D_{com}$ is the compressor investment in thousand $/a; $D_{mem}$ is the membrane separator depreciation cost in thousand $/a; $D_{PSA}$ is the PSA depreciation cost in thousand $/a; $D_{EHP}$ is the EHP depreciation cost in thousand $/a; and $D_{other}$ is the other equipment depreciation cost in thousand $/a.

The total operating cost (TOC) of helium recovery and purification can be calculated by Equation (A4):

(A4) $$TOC=C_{ele}+C_{cw}$$

where TOC is the total operating cost in thousand $/a; $C_{ele}$ is the electricity cost in thousand $/a; and $C_{cw}$ is the cooling water cost in thousand $/a.

The total annual cost (TAC) of helium recovery and purification can be calculated by Equation (A5):

(A5) TAC=DC+TOC

where TAC is the total annual cost, thousand $/a; is DC the depreciation cost, thousand $/a; TOC is the total operating cost, thousand $/a.

Finally, the cost of helium production can be calculated by Equation (A6):

(A6) $$PC=\frac{TAC}{F_{He}}$$

where PC is the cost of helium production in $/kg; TAC is the total annual cost in thousand $/a; and $F_{He}$ is the annual helium yield in kg/a.
